# Poly[aqua­[μ_3_-4-carb­oxy-2-(pyridin-4-yl)-1*H*-imidazole-5-carboxyl­ato-κ^5^ *N* ^1^,*O* ^5^:*N* ^3^,*O* ^4^:*N* ^2^]nickel(II)]

**DOI:** 10.1107/S1600536812001900

**Published:** 2012-01-21

**Authors:** Xue-Min Jing, Shu-zhe Gong, Li-Wei Xiao

**Affiliations:** a Faculty of Chemistry and Material Science, Langfang Teachers College, Langfang, Hebei 065000, People’s Republic of China; b Key Laboratory of Oilfield Applied Chemistry, College of Heilongjiang Province, Chemistry & Chemical Engineering Daqing Normal University, Daqing, Heilongjiang 163712, People’s Republic of China

## Abstract

The water-coordinated Ni^2+^ cation in the title compound, [Ni(C_10_H_5_N_3_O_4_)(H_2_O)]_*n*_, assumes an octa­hedral NiN_3_O_3_ coord­ination mode and is *N*,*O*-chelated by two deprotonated 2-(pyridin-4-yl)-1*H*-imidazole-4,5-dicarb­oxy­lic acid (HPyImDC^2−^) ligands, forming a layer structure extending in the *bc* plane. The chains are arranged along the *b*-axis direction, forming a layer structure extending in the *bc* plane. O—H⋯O hydrogen bonding between the layers results in the formation of a three-dimensional supra­molecular framework. The structure is isotypic with the Zn analogue [Li *et al.* (2009). *Cryst. Growth Des.* **6**, 3423–3431].

## Related literature

For the isotypic Zn compound, see: Li *et al.* (2009 ▶). The HPyImDC^2−^ anion behaves as a T-shaped linker, see: Jing *et al.* (2010 ▶).
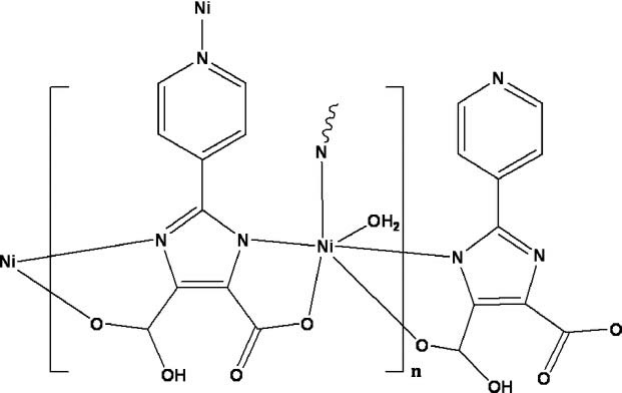


## Experimental

### 

#### Crystal data

[Ni(C_10_H_5_N_3_O_4_)(H_2_O)]
*M* *_r_* = 307.88
Monoclinic, 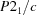

*a* = 7.5117 (15) Å
*b* = 11.400 (2) Å
*c* = 12.896 (4) Å
β = 109.04 (3)°
*V* = 1043.9 (4) Å^3^
*Z* = 4
Mo *K*α radiation
μ = 1.88 mm^−1^
*T* = 293 K
0.21 × 0.16 × 0.13 mm

#### Data collection

Bruker SMART CCD area-detector diffractometer
Absorption correction: multi-scan (*SADABS*; Sheldrick, 1996 ▶) *T*_min_ = 0.216, *T*_max_ = 0.422
10075 measured reflections
2377 independent reflections
1951 reflections with *I* > 2σ(*I*)
*R*_int_ = 0.067

#### Refinement

*R*[*F* ^2^ > 2σ(*F* ^2^)] = 0.051
*wR*(*F* ^2^) = 0.118
*S* = 1.04
2377 reflections
200 parameters
H atoms treated by a mixture of independent and constrained refinement
Δρ_max_ = 0.55 e Å^−3^
Δρ_min_ = −1.09 e Å^−3^

### 

Data collection: *SMART* (Bruker, 2002 ▶); cell refinement: *SAINT* (Bruker, 2002 ▶); data reduction: *SAINT*; program(s) used to solve structure: *SHELXS97* (Sheldrick, 2008 ▶); program(s) used to refine structure: *SHELXL97* (Sheldrick, 2008 ▶); molecular graphics: *XP* in *SHELXTL* (Sheldrick, 2008 ▶); software used to prepare material for publication: *SHELXTL*.

## Supplementary Material

Crystal structure: contains datablock(s) global, I. DOI: 10.1107/S1600536812001900/hp2024sup1.cif

Structure factors: contains datablock(s) I. DOI: 10.1107/S1600536812001900/hp2024Isup2.hkl

Supplementary material file. DOI: 10.1107/S1600536812001900/hp2024Isup4.cdx

Additional supplementary materials:  crystallographic information; 3D view; checkCIF report

## Figures and Tables

**Table 1:** Hydrogen-bond geometry (Å, °)

*D*—H⋯*A*	*D*—H	H⋯*A*	*D*⋯*A*	*D*—H⋯*A*
O1—H1⋯O3	0.94 (7)	1.57 (7)	2.501 (4)	171 (7)
O1*W*—H1*A*⋯O3^i^	0.78 (9)	1.95 (9)	2.726 (5)	174 (9)
O1*W*—H1*B*⋯O1^ii^	0.73 (6)	2.35 (6)	3.007 (5)	150 (5)

Symmetry codes: (i) 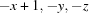; (ii) 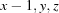.

**Fig. 1.**
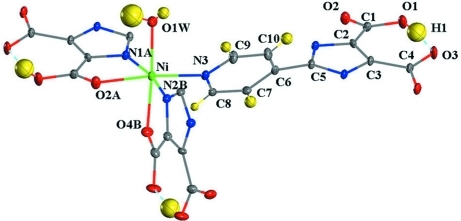
A view of the centrosymmetric molecule of (I), with displacement ellipsoids drawn at the 25% probability level [symmetry code: (i) -x, -y - 1, -z; (ii) x, -y - 1/2, z + 1/2; (iii) x, -y - 1/2, z - 1/2]

**Fig. 2.**
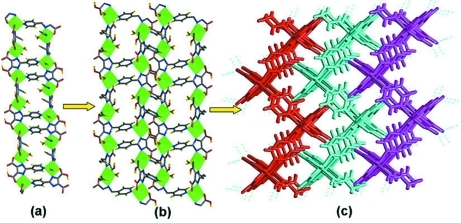
(a) A view showing the one-dimensional (one-dimensional) chain along the c direction; (b) one-dimensional chains arranged in the b direction to be a two-dimensional layer structure; (c) the two-dimensional layers packed in an AAA way via hydrogen-bonding interactions to be a three-dimensional network.

## supplementary crystallographic information

### Comment

Li *et al.* (2009) described the structure of [Zn(C_10_H_5_N_3_O_4_)H_2_O] as a stairway-like two-dimensional 3,3-connected layer held together *via* hydrogen-bonding interactions involving the carboxylic acid and water H atoms to be a three-dimensional network. The HPyImDC^2-^ anion behaves as a T-shaped linker (Jing *et al.*, 2010) with one N atoms and bis-N,*O*-bridging modes chelating the Ni(II) atoms. The present centrosymmetric Ni analogue, (Fig. 1) is isomorphous, the two compounds having nearly identical unit-cell parameters.

As shown in Fig. 2a, the {NiN_3_O_3_} octahedra connect with the T-shaped HPyImDC^2-^ anions to be a one-dimensional chain structure extending in the c direction. Then these one-dimensional chains arrange along the b direction to be a two-dimensional layer structure extending in the *bc* plane (Fig. 2 b), which are further connected through the hydrogen bonds occurred between O(1 W)—H(1 A)···O(3) (-*x* + 1,-*y*,-*z*) and O(1 W)—H(1B)···O(1)(*x*-1, *y*, *z*), respectively, to construct a three-dimensional supramolecular framework (Fig. 2c and Table 1).

### Experimental

Preparation of the complex.

A solution of NiCl_2_6H_2_O (0.012 g, 0.5 mmol) and H_3_PyImDC (0.012 g, 0.05 mmol) in DMF (1 ml) and H_2_O (0.5 ml) was sealed into a 15 ml Teflon-lined stainless autoclave and heated at 433 K for 4 days and then cooled to room temperature gradually to afford well formed green block crystals in about 60% yield (based on Zn). Elemental analysis found (%): C, 39.06; H, 2.30; N, 13.72; Ni, 19.01. H_7_C_10_N_3_O_5_Ni requires (%): C, 39.01; H, 2.29; N, 13.65; Ni, 19.06. IR (KBr, cm^-1^): 3571 (*s*), 3083 (*m*), 2560 (w), 1675 (w), 1565 (*versus*), 1271 (*s*), 842 (*m*), 567 (w).

### Refinement

The H atoms bonded to C were positioned geometrically with C—H distance 0.93–0.96 Å, and treated as riding atoms, with *U*_iso_(H)=1.1Ueq(C). The H atoms bonded to O were located in a difference Fourier map and refined isotropically.

### Crystal data

**Table:**

[Ni(C_10_H_5_N_3_O_4_)(H_2_O)]	*D*_x_ = 1.959 Mg m^−^^3^
*M**_r_* = 307.88	Melting point: not measured K
Monoclinic, *P*2_1_/*c*	Mo *K*α radiation, λ = 0.71073 Å
*a* = 7.5117 (15) Å	Cell parameters from 2377 reflections
*b* = 11.400 (2) Å	θ = 3.3–27.4°
*c* = 12.896 (4) Å	µ = 1.88 mm^−^^1^
β = 109.04 (3)°	*T* = 293 K
*V* = 1043.9 (4) Å^3^	Block, green
*Z* = 4	0.21 × 0.16 × 0.13 mm
*F*(000) = 624	

### Data collection

**Table:**

Bruker SMART CCD area-detector diffractometer	2377 independent reflections
Radiation source: fine-focus sealed tube	1951 reflections with *I* > 2σ(*I*)
graphite	*R*_int_ = 0.067
Detector resolution: 9.00cm pixels mm^-1^	θ_max_ = 27.4°, θ_min_ = 3.3°
phi and ω scans	*h* = −9→9
Absorption correction: multi-scan (*SADABS*; Sheldrick, 1996)	*k* = −14→14
*T*_min_ = 0.216, *T*_max_ = 0.422	*l* = −16→16
10075 measured reflections	

### Refinement

**Table:**

Refinement on *F*^2^	Primary atom site location: structure-invariant direct methods
Least-squares matrix: full	Secondary atom site location: difference Fourier map
*R*[*F*^2^ > 2σ(*F*^2^)] = 0.051	Hydrogen site location: inferred from neighbouring sites
*wR*(*F*^2^) = 0.118	H atoms treated by a mixture of independent and constrained refinement
*S* = 1.04	*w* = 1/[σ^2^(*F*_o_^2^) + (0.0468*P*)^2^ + 3.3079*P*] where *P* = (*F*_o_^2^ + 2*F*_c_^2^)/3
2377 reflections	(Δ/σ)_max_ < 0.001
200 parameters	Δρ_max_ = 0.55 e Å^−^^3^
0 restraints	Δρ_min_ = −1.09 e Å^−^^3^

### Special details

**Table:**

Geometry. All e.s.d.'s (except the e.s.d. in the dihedral angle between two l.s. planes) are estimated using the full covariance matrix. The cell e.s.d.'s are taken into account individually in the estimation of e.s.d.'s in distances, angles and torsion angles; correlations between e.s.d.'s in cell parameters are only used when they are defined by crystal symmetry. An approximate (isotropic) treatment of cell e.s.d.'s is used for estimating e.s.d.'s involving l.s. planes.
Refinement. Refinement of *F*^2^ against ALL reflections. The weighted *R*-factor *wR* and goodness of fit *S* are based on *F*^2^, conventional *R*-factors *R* are based on *F*, with *F* set to zero for negative *F*^2^. The threshold expression of *F*^2^ > σ(*F*^2^) is used only for calculating *R*-factors(gt) *etc*. and is not relevant to the choice of reflections for refinement. *R*-factors based on *F*^2^ are statistically about twice as large as those based on *F*, and *R*- factors based on ALL data will be even larger.

### Fractional atomic coordinates and isotropic or equivalent isotropic displacement parameters (Å^2^)

**Table:**

	*x*	*y*	*z*	*U*_iso_*/*U*_eq_	
Ni1	0.32031 (7)	−0.26730 (4)	0.18242 (4)	0.01465 (17)	
O1	0.7267 (4)	−0.0698 (3)	0.1164 (3)	0.0285 (7)	
H1	0.740 (10)	−0.063 (6)	0.046 (6)	0.08 (2)*	
O2	0.5375 (4)	−0.1450 (3)	0.1996 (2)	0.0234 (7)	
O3	0.7296 (4)	−0.0603 (3)	−0.0768 (2)	0.0275 (7)	
O4	0.5464 (4)	−0.1211 (2)	−0.2426 (2)	0.0210 (6)	
O1W	0.1140 (5)	−0.1417 (3)	0.1203 (3)	0.0243 (7)	
H1A	0.163 (12)	−0.087 (8)	0.106 (7)	0.11 (3)*	
H1B	0.041 (8)	−0.120 (5)	0.142 (4)	0.030 (16)*	
N1	0.3422 (5)	−0.2756 (3)	0.0217 (3)	0.0158 (7)	
N2	0.3437 (5)	−0.2632 (3)	−0.1527 (2)	0.0141 (6)	
N3	−0.1352 (5)	−0.5920 (3)	−0.1621 (3)	0.0168 (7)	
C1	0.5860 (6)	−0.1354 (3)	0.1176 (3)	0.0178 (8)	
C2	0.4806 (5)	−0.1986 (3)	0.0175 (3)	0.0142 (8)	
C3	0.4813 (5)	−0.1914 (3)	−0.0886 (3)	0.0148 (8)	
C4	0.5922 (6)	−0.1201 (3)	−0.1406 (3)	0.0172 (8)	
C5	0.2647 (5)	−0.3130 (3)	−0.0826 (3)	0.0144 (8)	
C6	0.1198 (5)	−0.4054 (3)	−0.1148 (3)	0.0151 (8)	
C7	−0.0568 (6)	−0.3944 (3)	−0.1030 (3)	0.0161 (8)	
H7	−0.090 (7)	−0.323 (4)	−0.074 (4)	0.029 (13)*	
C8	−0.1783 (6)	−0.4892 (4)	−0.1272 (3)	0.0175 (8)	
H8	−0.297 (6)	−0.478 (4)	−0.122 (3)	0.014 (10)*	
C9	0.0311 (6)	−0.6007 (4)	−0.1785 (4)	0.0231 (9)	
H9	0.047 (6)	−0.676 (4)	−0.206 (4)	0.023 (12)*	
C10	0.1607 (6)	−0.5112 (4)	−0.1567 (3)	0.0222 (9)	
H10	0.285 (7)	−0.522 (4)	−0.161 (4)	0.030 (13)*	

### Atomic displacement parameters (Å^2^)

**Table:**

	*U*^11^	*U*^22^	*U*^33^	*U*^12^	*U*^13^	*U*^23^
Ni1	0.0168 (3)	0.0167 (3)	0.0120 (3)	−0.0020 (2)	0.00689 (19)	−0.0009 (2)
O1	0.0247 (17)	0.0394 (19)	0.0202 (16)	−0.0164 (14)	0.0058 (13)	−0.0018 (14)
O2	0.0272 (16)	0.0310 (16)	0.0141 (14)	−0.0091 (13)	0.0097 (12)	−0.0055 (12)
O3	0.0250 (16)	0.0351 (18)	0.0218 (15)	−0.0181 (14)	0.0068 (13)	0.0018 (14)
O4	0.0247 (16)	0.0259 (15)	0.0155 (14)	−0.0076 (12)	0.0107 (12)	0.0026 (12)
O1W	0.0235 (18)	0.0257 (17)	0.0274 (17)	0.0037 (14)	0.0134 (14)	0.0046 (14)
N1	0.0180 (16)	0.0177 (16)	0.0133 (15)	−0.0024 (14)	0.0074 (13)	0.0009 (13)
N2	0.0172 (16)	0.0152 (15)	0.0126 (15)	−0.0023 (13)	0.0085 (13)	0.0000 (13)
N3	0.0197 (17)	0.0187 (16)	0.0124 (15)	−0.0032 (14)	0.0059 (13)	0.0012 (13)
C1	0.018 (2)	0.021 (2)	0.0136 (19)	−0.0036 (16)	0.0045 (16)	0.0010 (16)
C2	0.0104 (18)	0.0183 (19)	0.0136 (18)	−0.0043 (14)	0.0034 (14)	−0.0016 (15)
C3	0.0158 (19)	0.0160 (18)	0.0151 (18)	−0.0024 (15)	0.0087 (15)	0.0015 (15)
C4	0.0165 (19)	0.020 (2)	0.018 (2)	−0.0020 (15)	0.0091 (16)	0.0035 (16)
C5	0.016 (2)	0.0146 (18)	0.0146 (18)	−0.0030 (15)	0.0078 (15)	−0.0009 (15)
C6	0.017 (2)	0.0199 (19)	0.0086 (17)	−0.0038 (16)	0.0054 (14)	0.0005 (15)
C7	0.017 (2)	0.0147 (19)	0.0164 (19)	0.0026 (15)	0.0059 (15)	0.0001 (16)
C8	0.014 (2)	0.023 (2)	0.0166 (19)	0.0025 (16)	0.0065 (15)	0.0073 (16)
C9	0.028 (2)	0.019 (2)	0.027 (2)	−0.0057 (18)	0.0151 (18)	−0.0062 (18)
C10	0.022 (2)	0.026 (2)	0.024 (2)	−0.0044 (17)	0.0150 (18)	−0.0045 (18)

### Geometric parameters (Å, °)

**Table:**

Ni1—O1W	2.070 (3)	N2—C3	1.365 (5)
Ni1—N3^i^	2.082 (3)	N2—Ni1^iii^	2.105 (3)
Ni1—O4^ii^	2.089 (3)	N3—C8	1.332 (5)
Ni1—O2	2.103 (3)	N3—C9	1.338 (5)
Ni1—N2^ii^	2.105 (3)	N3—Ni1^i^	2.082 (3)
Ni1—N1	2.134 (3)	C1—C2	1.465 (5)
O1—C1	1.299 (5)	C2—C3	1.372 (5)
O1—H1	0.94 (7)	C3—C4	1.474 (5)
O2—C1	1.230 (5)	C5—C6	1.474 (5)
O3—C4	1.285 (5)	C6—C7	1.390 (5)
O4—C4	1.246 (5)	C6—C10	1.396 (6)
O4—Ni1^iii^	2.089 (3)	C7—C8	1.384 (6)
O1W—H1A	0.78 (9)	C7—H7	0.96 (5)
O1W—H1B	0.73 (6)	C8—H8	0.93 (4)
N1—C5	1.349 (5)	C9—C10	1.374 (6)
N1—C2	1.375 (5)	C9—H9	0.95 (5)
N2—C5	1.356 (5)	C10—H10	0.96 (5)
			
O1W—Ni1—N3^i^	95.68 (14)	O2—C1—O1	122.1 (4)
O1W—Ni1—O4^ii^	173.34 (13)	O2—C1—C2	119.2 (3)
N3^i^—Ni1—O4^ii^	89.89 (13)	O1—C1—C2	118.7 (3)
O1W—Ni1—O2	92.26 (14)	C3—C2—N1	109.0 (3)
N3^i^—Ni1—O2	170.92 (13)	C3—C2—C1	132.3 (3)
O4^ii^—Ni1—O2	82.49 (12)	N1—C2—C1	118.5 (3)
O1W—Ni1—N2^ii^	94.71 (13)	N2—C3—C2	108.6 (3)
N3^i^—Ni1—N2^ii^	94.98 (12)	N2—C3—C4	118.9 (3)
O4^ii^—Ni1—N2^ii^	81.13 (11)	C2—C3—C4	132.4 (4)
O2—Ni1—N2^ii^	88.74 (12)	O4—C4—O3	124.6 (4)
O1W—Ni1—N1	86.54 (13)	O4—C4—C3	118.2 (3)
N3^i^—Ni1—N1	95.94 (12)	O3—C4—C3	117.1 (3)
O4^ii^—Ni1—N1	96.54 (12)	N1—C5—N2	113.1 (3)
O2—Ni1—N1	80.11 (11)	N1—C5—C6	123.1 (3)
N2^ii^—Ni1—N1	168.83 (12)	N2—C5—C6	123.7 (3)
C1—O1—H1	114 (4)	C7—C6—C10	117.3 (4)
C1—O2—Ni1	113.9 (3)	C7—C6—C5	123.2 (4)
C4—O4—Ni1^iii^	113.4 (2)	C10—C6—C5	119.4 (3)
Ni1—O1W—H1A	107 (6)	C8—C7—C6	119.2 (4)
Ni1—O1W—H1B	130 (4)	C8—C7—H7	121 (3)
H1A—O1W—H1B	107 (7)	C6—C7—H7	120 (3)
C5—N1—C2	104.4 (3)	N3—C8—C7	123.3 (4)
C5—N1—Ni1	146.9 (3)	N3—C8—H8	119 (3)
C2—N1—Ni1	108.0 (2)	C7—C8—H8	117 (3)
C5—N2—C3	104.9 (3)	N3—C9—C10	123.2 (4)
C5—N2—Ni1^iii^	146.2 (3)	N3—C9—H9	111 (3)
C3—N2—Ni1^iii^	108.1 (2)	C10—C9—H9	126 (3)
C8—N3—C9	117.5 (3)	C9—C10—C6	119.4 (4)
C8—N3—Ni1^i^	119.6 (3)	C9—C10—H10	122 (3)
C9—N3—Ni1^i^	122.9 (3)	C6—C10—H10	118 (3)

Symmetry codes: (i) −*x*, −*y*−1, −*z*; (ii) *x*, −*y*−1/2, *z*+1/2; (iii) *x*, −*y*−1/2, *z*−1/2.

### Hydrogen-bond geometry (Å, °)

**Table:**

*D*—H···*A*	*D*—H	H···*A*	*D*···*A*	*D*—H···*A*
O1—H1···O3	0.94 (7)	1.57 (7)	2.501 (4)	171 (7)
O1W—H1A···O3^iv^	0.78 (9)	1.95 (9)	2.726 (5)	174 (9)
O1W—H1B···O1^v^	0.73 (6)	2.35 (6)	3.007 (5)	150 (5)

Symmetry codes: (iv) −*x*+1, −*y*, −*z*; (v) *x*−1, *y*, *z*.

## References

[bb1] Bruker (2002). *SMART* and *SAINT* Bruker AXS Inc., Madison, Wisconsin, USA.

[bb2] Jing, X., Meng, H., Li, G., Yu, Y., Huo, Q., Eddaoudi, M. & Liu, Y. (2010). *Cryst. Growth Des.* **10**, 3489–3495.

[bb3] Li, X., Wu, B., Niu, C., Niu, Y. & Zhang, H. (2009). *Cryst. Growth Des.* **9**, 3423–3431.

[bb4] Sheldrick, G. M. (1996). *SADABS* University of Göttingen, Germany.

[bb5] Sheldrick, G. M. (2008). *Acta Cryst.* A**64**, 112–122. 10.1107/S0108767307043930 18156677

